# Exposure of Perfluorinated Chemicals through Lactation: Levels of Matched Human Milk and Serum and a Temporal Trend, 1996–2004, in Sweden

**DOI:** 10.1289/ehp.9491

**Published:** 2006-11-28

**Authors:** Anna Kärrman, Ingrid Ericson, Bert van Bavel, Per Ola Darnerud, Marie Aune, Anders Glynn, Sanna Lignell, Gunilla Lindström

**Affiliations:** 1 Man–Technology–Environment (MTM) Research Centre, Örebro University, Örebro, Sweden; 2 National Food Administration, Uppsala, Sweden

**Keywords:** human exposure, LC-MS, PFC, PFOA, PFOS

## Abstract

**Background:**

Only limited data exist on lactation as an exposure source of persistent perfluorinated chemicals (PFCs) for children.

**Objectives:**

We studied occurrence and levels of PFCs in human milk in relation to maternal serum together with the temporal trend in milk levels between 1996 and 2004 in Sweden. Matched, individual human milk and serum samples from 12 primiparous women in Sweden were analyzed together with composite milk samples (25–90 women/year) from 1996 to 2004.

**Results:**

Eight PFCs were detected in the serum samples, and five of them were also above the detection limits in the milk samples. Perfluorooctanesulfonate (PFOS) and perfluorohexanesulfonate (PFHxS) were detected in all milk samples at mean concentrations of 0.201 ng/mL and 0.085 ng/mL, respectively. Perfluorooctanesulfonamide (PFOSA), perfluorooctanoic acid (PFOA), and perfluorononanoic acid (PFNA) were detected less frequently.

**Discussion:**

The total PFC concentration in maternal serum was 32 ng/mL, and the corresponding milk concentration was 0.34 ng/mL. The PFOS milk level was on average 1% of the corresponding serum level. There was a strong association between increasing serum concentration and increasing milk concentration for PFOS (*r*^2^ = 0.7) and PFHxS (*r*^2^ = 0.8). PFOS and PFHxS levels in composite milk samples were relatively unchanged between 1996 and 2004, with a total variation of 20 and 32% coefficient of variation, respectively.

**Conclusion:**

The calculated total amount of PFCs transferred by lactation to a breast-fed infant in this study was approximately 200 ng/day. Lactation is a considerable source of exposure for infants, and reference concentrations for hazard assessments are needed.

An increasing number of studies show that humans are exposed to a large number of perfluorinated chemicals (PFCs), including perfluorooctane sulfonate (PFOS) (Calafat et al. 2006b; Karrman et al. 2006b; Yeung et al. 2006). Pooled serum from Australians < 16 years of age (median age 11–13 years) had equal or higher levels of several PFCs compared with adults (Karrman et al. 2006a). PFOS concentration in 2- to 12-year-old children in the United States ranged between 6.7 and 515 ng/mL, and the mean concentration of PFOS and additional PFCs exceeded those for adult and elderly populations (Olsen et al. 2004). Exposure routes for children therefore need to be assessed.

The surfactant properties of perfluorinated chemicals have made them desirable for extensive use in fabric, leather, and apparel treatment, in protection of food packaging and paper products, and in fire-extinguishing foam and insecticides. The inertness and great heat stability of these chemicals have also extended their industrial use.

PFCs have been shown to be persistent, to biomagnify, and to be transported into remote regions (Giesy and Kannan 2001; Smithwick et al. 2005; Tomy et al. 2004). They are potentially toxic for the environment and humans (3M 2003; Kennedy et al. 2004). Several studies have reported the increase of PFC levels in humans and wildlife up to the late 1990s (Harada et al. 2004; Holmström et al. 2004; Olsen et al. 2005b). Recent efforts by authorities and manufacturing companies to phase out the production and reduce emissions of PFCs has not yet been observed as declining environmental concentrations.

Prenatal as well as postnatal toxicity of PFOS was observed in rat and mouse, including increased liver weights, growth lags, and delayed development (Lau et al. 2003; Thibodeaux et al. 2003). Reduced weight gain and delayed development were also observed for perfluorooctanoic acid (PFOA) (Lau et al. 2004). However, assessment of risks for humans by extrapolating animal data must take into consideration several unknown pharmacokinetic parameters, such as species-specific clearance (Hanhijärvi et al. 1982; Organisation for Economic Co-operation and Development 2002).

Prenatal exposure and transfer of several PFCs through lactation has been confirmed for humans. Cord blood concentrations of PFOS were on average three times lower (1.6–5.3 ng/mL) than the corresponding maternal blood concentration from nonoccupational-exposed Japanese women (Inoue et al. 2004). A study on two human milk samples from the United States was the first to show the presence of PFCs in human milk (Kuklenyik et al. 2004). Recently So et al. (2006) reported that low levels (10–592.6 pg/mL) of PFOS, PFOA, perfluorohexane sulfonate (PFHxS), and perfluorononanoic acid (PFNA) were found in human milk of women living in China. There is to our knowledge no information about the degree of maternal PFCs transferred to the infant through lactation. A cross-fostering study on Sprague-Dawley rats indicated that PFOS is transferred to pups through lactation with a milk concentration 10–100 times lower than the maternal serum concentration (Kuklenyik et al. 2004). The concentration of PFOA in rat’s milk was estimated to be 10 times lower than the concentration in maternal plasma (Hinderliter et al. 2005). The specific transfer mechanisms are however not clear.

The objective of this study was to elucidate the relationship between maternal PFC serum levels and breast milk levels to better understand the lactational transfer of PFCs. For this purpose, matched individual milk and serum samples from 12 Swedish primiparous women were collected in 2004. In addition, the trend in Swedish breast milk PFC concentrations between 1996 and 2004 is also reported. This is the first report on the relationship of PFCs in human maternal serum and milk and the first temporal trend in breast milk PFC levels. Included in this study were four perfluoroalkylsulfonates (C4, C6, C8, C10), one polyfluorinated sulfonate (C8), seven perfluoroalkylcarboxylates (C6, C8, C9, C10, C11, C12, C14) and perfluorooctanesulfonamide (PFOSA).

## Materials and Methods

### Samples

Individual milk and serum samples from 12 women in Uppsala, Sweden, were collected in 2004. Milk samples from 25–90 women were collected each year between 1996 and 2004 and pooled into an annual composite sample. Donors originated from four regions in Sweden (Uppsala, Lund, Göteborg, Lycksele). All samples were from primiparous women and were collected in glass bottles during the third week after delivery and stored in plastic containers at −20°C. A summary of the sample information, including age of donors and number of pooled samples, is given in Table 1. The local ethics committee approved the design of this study, and informed consent was obtained from the study participants.

### Chemicals

We purchased ammonium acetate [> 99%, for high-performance liquid chromatography (HPLC)] from Fluka (Steinheim, Germany), formic acid (98–100%) from Scharlau (Barcelona, Spain), and methanol (HPLC) from Labscan (Dublin, Ireland). All water used was laboratory-produced ultrapure water. Ammonium hydroxide (25% in water) and sodium acetate were purchased from Merck (Darmstadt, Germany). Perfluorobutanesulfonate (PFBuS) tetrabutylammonium salt (> 98%), PFOS potassium salt (> 98%), perfluorodecanoic acid (PFDA; > 97%), and perfluorohexanoic acid (PFHxA; > 97%) were purchased from Fluka. Perfluoroheptanoic acid (PFHpA; 99%), PFNA (97%), PFOA (96%), perfluorodecanesulfonate (PFDS) ammonium salt [25% weight in 2-butoxyethanol (37%) in water], perfluoroundecanoic acid (PFUnDA; 95%), and perfluorotetradecanoic acid (PFTDA; 97%) were purchased from Aldrich (Steinheim, Germany, and Milwaukee, WI, USA). PFOSA (97%) and *7H*-PFHpA (98%) were purchased from ABCR (Karlsruhe, Germany). *1H,1H,2H,2H*-PFOS (THPFOS, purity unknown), and PFHxS (98%) were purchased from Interchim (Montlucon, France). ^13^C_4_-Labeled PFOA, 13C_4_-labeled PFOS, and ^13^C_5_-labeled PFNA were from Wellington Laboratories (Guelph, Ontario, Canada).

### Extraction

The serum and milk samples were extracted using weak anion exchange, solid-phase extraction (Waters Oasis WAX, Milford, MA, USA) based on the method by Taniyasu and colleagues (Taniyasu et al. 2005). Internal standards (^13^C_4_-PFOA and ^13^C_4_-PFOS) and 2 mL formic acid/water (1:1) were added to 1 mL milk and 0.5 mL serum. The solution was sonicated for 15 min and centrifuged at 10,000 × *g* for 30 min. The supernatant was extracted and the perfluorinated compounds were eluted with 1 mL 2% ammonium hydroxide in methanol, after washing the column with 2 mL sodium acetate buffer solution, pH 4, and 2 mL 40% methanol in water. Sodium acetate buffer was not used for the serum samples. The final volume for the serum extracts was 500 μL. Milk extracts were further evaporated to 30 μL, and 20 μL 2-mM ammonium acetate in water was added. Finally, filtration through a Microcon YM-3 centrifugal filter (Millipore, Billerica, MA, USA) was conducted at 14,000 × *g* for 30 min. Performance standards, ^13^C_5_-PFNA and *7H*-PFHpA, were added to both milk and serum extracts immediately before injection.

### Analysis

We performed the analysis using an Agilent 1100 HPLC system coupled to an Agilent 1100 mass spectrometric detector (Agilent, Waldbronn, Germany) with an atmospheric electrospray interface operating in negative ion mode. Separation was performed on a Discovery HS C18 (50 mm length, 2.1 mm inner diameter, 3 μm particles, 120 Å pore size) column with a guard column of the same material (20 mm length, 2.1 mm inner diameter, 3 μm particles, 120 Å pore size) (Supelco, Bellefonte PA, USA). Both columns were kept at 40°C. An extra guard column (HyperCarb graphitic carbon, 4 mm length, 10 mm inner diameter, 5 μm particle size; Termo Hypersil-Keystone, Bellefonte PA, USA) was inserted between the pump and injector to remove any fluorochemicals originating from the HPLC system. Injection volume was 10 μL and the flow rate was set to 300 μL/min. The mobile phases consisted of 2 mM ammonium acetate in methanol and 2 mM ammonium acetate in water. HPLC gradient and MS settings have been described in detail elsewhere (Karrman et al. 2005).

### Quality assurance

Quantification was performed using the internal standard method with standards dissolved in 35% methanol in water. We used ^13^C_4_-PFOS as internal standard for the sulfonates and PFOSA, and ^13^C_4_-PFOA for the carboxylates. We used ^13^C_5_-PFNA and *7H*-PFHpA to monitor the recovery of the internal standards. The recovery was on average 67%, within 50–130% for 78% of all milk samples and within 84–97% for all serum samples. An overview of the method performance is given in Table 2. Recoveries were evaluated by three or five replicate fortifications to a low-contaminated serum sample and a breast milk sample containing PFCs below the detection limit. Average recoveries were > 50% for all compounds except for PFOSA, PFDA, PFUnDA, and PFDoDA (34–47%) and the coefficient of variation (CV) was 2–27% for multiple determinations. Containers used for storage of milk and serum samples were extracted with methanol and did not show traces of the target compounds. Procedural blank trace levels were detected for PFOA, PFOS, and PFNA (Table 3). In the case of blank levels, the mean blank signal plus 3 SDs of multiple blank injections were subtracted from the calculated concentrations in the samples. A blank corrected concentration was reported provided that the blank level was ≤50% of the uncorrected concentration. Detection limits for serum and breast milk were 0.1–1.1 ng/mL and 0.005–0.209 ng/mL, respectively. The selectivity of the mass spectrometry (MS) analysis was verified by triple quadrupole mass spectrometry (MS/MS) analysis. All breast milk samples (1 mL) were extracted in duplicates. The first sample extract was evaporated to 50 μL and injected on the LC-MS system (10 μL). The second extract was kept at 500 μL, of which 200 μL was injected on a column-switching LC system connected to a triple quadrupole MS system (Micromass QuattroII, Altrincham, UK). Further quality assurance was taken by successful participation in the first interlaboratory study on PFCs (Van Leeuwen et al. 2006).

## Results

A summary of the results of 12 individual matched milk and serum samples is given in Table 3. Highest mean serum concentration was obtained for PFOS (20.7 ng/mL) followed by PFHxS (4.7 ng/mL), PFOA (3.8 ng/mL), PFNA (0.80 ng/mL), PFDA (0.53 ng/mL), PFUnDA (0.40 ng/mL), and PFOSA (0.24 ng/mL). PFDS was detected in only one serum sample (0.33 ng/mL). Of the eight PFCs found in the serum samples, five were detected in the matched milk samples at the current detection limits. PFOS and PFHxS were detected in all milk samples at mean concentrations of 0.201 ng/mL and 0.085 ng/mL, respectively. PFOSA was detected in eight milk samples with a mean concentration of 0.013 ng/mL, and PFNA was detected in two milk samples (0.020 and 0.014 ng/mL). Similar PFC occurrence and levels were found in the milk composite samples collected during the 8 years between 1996 and 2004 (Table 4).

**Figure 1:**
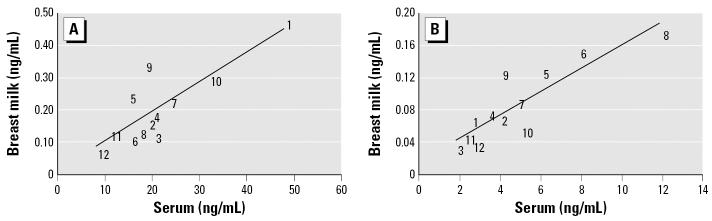
Scatterplot and regression analysis of levels in matched serum and milk samples from 12 Swedish primiparous women (numbered 1–12), 2004. (*A*) PFOS (M:S ratio, 0.01:1; CV, 38%; *y* = 0.0092*x* + 0.0104; *r*^2^ = 0.6868). (*B*) PFOA (M:S ratio, 0.02:1; CV, 28%; *y* = 0.0144*x* + 0.0178; *r*^2^ = 0.7707).

**Figure 2:**
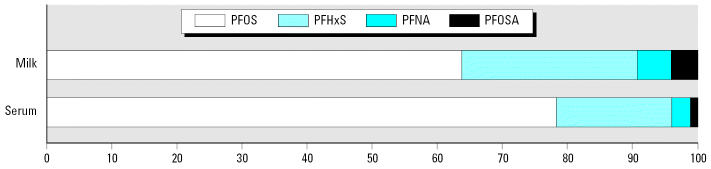
The average contribution (%) of PFOS, PFHxS, PFNA, and PFOSA to the total concentration in 12 matched milk and serum samples from primiparous women in Sweden, 2004.

Milk levels were lower compared with the matched serum levels on a volume basis (nanograms per milliliter). The mean ratio between milk and serum (M:S) concentration was 0.01:1 for PFOS, 0.02:1 for PFHxS, and 0.07:1 for PFOSA (Table 3). The M:S ratios for PFOA and PFNA are uncertain because only one and two milk samples, respectively, contained levels above the detection limit. Simple regression analysis and the Spearman’s correlation test of the matched serum and milk samples show a significant association (*r*^2^ = 0.7–0.8, *p* < 0.05) between levels of PFOS and PFHxS in serum and milk (Figure 1). The percentage contribution of each of PFHxS, PFOS, PFOSA, and PFNA to the total concentration in serum and milk is given in Figure 2.

The limits of detection (LOD) in human milk were between 0.005 and 0.010 ng/mL, except for PFHxA and PFHpA, which were an order of magnitude higher (0.1 ng/mL) (Table 2). A relatively high blank level was obtained for PFOA (0.209 ng/mL). PFOA is reported in only one milk sample as a consequence of the high blank level and the quantification criteria.

## Discussion

The serum levels in the present study are similar to or lower than the levels found in a previous study on 17 Swedish human plasma samples collected in 1998–2000 from men and women (Karrman et al. 2004). The Swedish PFOS and PFOA blood levels are similar to levels in, for example, Canada, Australia, and some less-industrialized regions in Japan, but somewhat lower than reported blood levels in the United States (Harada et al. 2004; Karrman et al. 2006a; Kubwabo et al. 2004; Olsen et al. 2003).

Only one study originating from China has previously reported levels of several PFCs in human milk. The Swedish levels are comparable to human milk from China except for those for PFOSA, which was not included in the Chinese study (So et al. 2006). In addition to detected PFCs in the present study, PFHpA, PFDA, and PFUnDA were also found in human milk from China.

PFOSA was frequently detected in the milk samples, unlike PFNA, PFDA and PFUnDA, even though the latter were detected at higher concentrations in the serum samples. This is most likely caused by the fact that PFOSA concentrations in plasma have been found to be only about 20% of the whole blood concentration on a volume basis (Karrman et al. 2006b). The total blood concentration of PFOSA available for excretion to milk is therefore about five times higher than the measured concentration in serum. The M:S ratio for PFOSA should therefore be close to that of PFOS if nearly all PFOSA were distributed to serum. The serum and milk pattern suggests that PFHxS is excreted to milk in a higher degree than PFOS and PFOSA. A preferential excretion of shorter, less hydrophobic PFCs is a possible explanation of the observed pattern, but could not be concluded in the present study because of the limited number of matched milk and serum samples.

The presented linear relationship between serum and milk levels suggests a partitioning process, which can be predicted from the PFC blood concentration on a volume basis. The steeper slope of PFHxS demonstrates the higher partition than PFOS to milk (Figure 1). The association between milk and serum concentrations could not be seen for PFOSA (*r*^2^ < 0.1). The PFOSA ratio between milk and serum can be influenced by several parameters. First, it has been suggested that PFOSA can degrade to PFOS in biologic systems (Tomy et al. 2003), which might affect the ratio between milk and serum. Second, PFOSA is partly lost during the separation of the red blood cells, which makes serum a poor matrix for determining PFOSA blood concentrations. Finally, relatively more milk and serum samples had levels of PFOSA close to the detection limit.

For more fat-soluble, persistent organohalogens, the levels in blood and milk are about the same when calculated on a fat basis and with a steady state assumption. On a volume basis, the ratio of lipophilic compounds in whole blood and milk is approximately 1:10, because of the higher lipid content in milk than in blood (Jensen and Slorach 1991). The lactational transfer of PFCs may be more similar to that of heavy metals. For example, the concentration of lead in milk has been found to be 5–10 times lower than that in blood (Jensen and Slorach 1991). Perfluorinated acids are generally believed to bind to serum albumin (Jones et al. 2003). It has been demonstrated that serum albumin in plasma has a large binding capacity for PFOA (6–9 binding sites per molecule and millimolar concentration in plasma) and the free fraction of PFOA in plasma was estimated to be < 5% (Han et al. 2003). The reason for the relatively higher PFC concentration in human serum than in milk is unknown.

Excretion of PFCs into milk may be accomplished by two ways that have been identified as transport mechanisms for chemical contaminants: binding to milk protein (protein content ~ 1 g/100 mL milk) or to the surface of fat (fat content ~ 4 g/100 mL milk) (Jensen and Slorach 1991). The fat content in milk fluctuates but does not vary significantly during the course of lactation, unlike the total protein content, which was shown to decrease rapidly during the first month of lactation. The serum albumin content of milk was, however, stable during a 6.5-month period of lactation (Lönnerdal et al. 1976). Assessing the amount of PFCs transferred and adsorbed by an infant during the course of lactation involves several assumptions—for instance, the variation of PFC concentration in milk with time and the uptake efficiency of PFCs from milk by the infant. The total mean PFC concentration of all detected compounds in the present study was 32 ng/mL in serum and 0.34 ng/mL in milk. Hypothetically, a lactation of 600 mL/day and 100% uptake would produce an exposure burden for an infant (and maternal excretion) of 203 ng PFCs per day, corresponding to 34 μg PFCs after 6 months, given a constant PFC concentration in milk during 6 months. A risk assessment is unfeasible because of the lack of human hazard assessment of each of the detected PFCs and of relevant reference intake levels or concentrations to compare with. However, So et al. (2006) used a reference dose (25 ng/kg/day) for PFOS estimated by the Environmental Working Group, based on the end point of increase in mammary fibroadenomas in a rat chronic toxicity study (Thayer and Houlihan 2002). Using the same assumptions (milk consumption 600 g/day, body weight 7 kg), two milk samples with the highest PFOS concentration in our study (0.465 and 0.337 ng/mL) exceed the reference dose and would therefore constitute a risk to the infant. However, there are several uncertainties that need clarification before any conclusions can be made.

This study contributes to PFC exposure risk assessments for infants, and the evaluation of lactation as an exposure pathway as well as a way for maternal excretion. Several studies indicate that females have lower blood concentrations of several PFCs than do males (Calafat et al. 2006a; Karrman et al. 2006a; Olsen et al. 2003). Elimination through lactation could be one explanation for this observation. However, a sex difference was observed also for 2- to 12-year-old children in the United States (Olsen et al. 2004).

**Figure 3:**
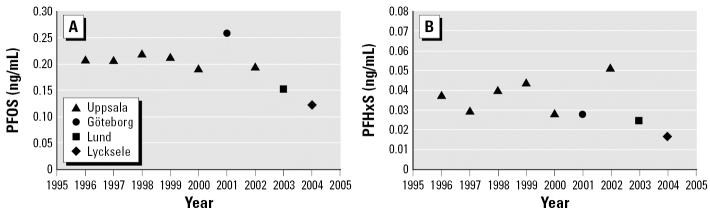
Temporal trend for (*A*) PFOS and (*B*) PFHxS in human composite milk samples from different regions in Sweden, 1996–2004.

PFOS and PFHxS were detected in composite milk samples collected each year between 1996 and 2003–2004 from four different regions in Sweden (Table 4). The variation of PFOS and PFHxS in the composite samples is remarkably small (a total variation of 20% and 32% CV, respectively), indicating that milk levels of PFOS and PFHxS have been constant in the last 8 years. Consequently, no clear temporal trend could be distinguished (Figure 3). However, the samples from 2001, 2003, and 2003–2004 were from regions different from the rest of the samples. Possible regional differences in human PFC levels in Sweden remain yet to be established. PFOS has been present in the Swedish environment at least since 1968, and the levels increased dramatically up to 1997 in guillemot eggs (Holmström et al. 2004). PFOS-related products were imported in Sweden until 2002 and will probably be used for a long period of time [KEMI (Swedish Chemicals Agency) 2004]. The global production of perfluorooctanesulfonyl fluoride started to decrease in 2001 after the phase-out decision by the major producer 3M (3M 2000). A possible effect of the actions taken by governments and producers in terms of declining environmental and human concentrations needs to be monitored for several years to come because of the persistence of PFCs [PFOS half-life is approximately 5 years in humans (Olsen et al. 2005a)].

The relatively low levels of PFCs present in the human milk samples challenged the analysis. By reducing the volume of the milk sample extracts by a factor of 100, required detection limits were achieved. As a consequence, traces in the procedural blanks were seen for several of the compounds monitored (Table 2). A confident quantification of PFOA in the milk samples was hampered by a high procedural blank contamination. PFOA is usually the second highest PFC found in human blood, except in Korea where PFOA levels have been reported to exceed those of PFOS (Kannan et al. 2004). PFOA contributed up to 36% of the total PFC content in human milk from China (So et al. 2006). The selectivity of the single quadrupole MS method was successfully verified with triple quadrupole MS/MS analysis. Qualitative comparison indicated that MS/MS analysis demonstrated on average 50% higher concentrations compared with the single quadrupole MS analysis. However, different preconcentration methods were used for the different instruments, and the differences seen between the methods can be multifactorial.

## Conclusions

The PFC level in human milk are about 1% of the corresponding level in serum. There is an indication that elimination of PFCs through lactation is compound-dependent and partitioning of PFCs into milk seems to relate to the concentration in maternal blood. A trend of PFC concentrations in milk between 1996 and 2004 could not be observed in the present study. Lactation is a considerable source of PFC exposure for infants. The present study indicates that approximately 200 ng PFCs per day may be transferred from a lactating mother to the infant. Reference concentrations as well as information on the infant’s uptake and excretion of PFCs during the lactation period are urgently needed for a full risk assessment. The ubiquitous presence and levels of PFOS in human milk justifies further monitoring of this class of contaminants in human milk worldwide.

## Correction

In the manuscript originally published online, in Table 2, some of the values for the blank concentrations of serum and milk were incorrect. Figure 1B describes PFOA, not PFHxS. These errors have been corrected here.

## Figures and Tables

**Table 1:** Overview of milk and serum samples, collected from primiparous women in Sweden.

Region	Year of collection	No.^a^	Age (years)^b^
Composite milk			
Uppsala	1996	25	29 (21–41)
Uppsala	1997	69	28 (21–38)
Uppsala	1998	90	29 (22–37)
Uppsala	1999	23	28 (22–36)
Uppsala	2000	30	30 (21–37)
Göteborg	2001	37	30 (19–40)
Uppsala	2002	31	30 (24–37)
Lund	2003	37	29 (25–39)
Lycksele	2003–2004	39	27 (20–36)
Individual milk and serum			
Uppsala	2004	—	29
Uppsala	2004	—	24
Uppsala	2004	—	29
Uppsala	2004	—	29
Uppsala	2004	—	28
Uppsala	2004	—	30
Uppsala	2004	—	31
Uppsala	2004	—	27
Uppsala	2004	—	33
Uppsala	2004	—	28
Uppsala	2004	—	22
Uppsala	2004	—	29

a Number of individual samples in the composite sample.

b Median (minimum–maximum).

**Table 2:** Performance of the methods for extracting human serum and milk samples.

	Percent of recovery (CV)		Detection limit (ng/mL)^a^		Blank concentration (ng/mL)^b^	
Compound	Serum (*n* = 5)	Milk (*n* = 3)	Serum	Milk	Serum	Milk
PFBuS	85 (2)	79 (4)	0.7	0.05	—	—
PFHxS	89 (3)	81 (3)	0.2	0.01	—	—
PFOS	82 (5)	83 (3)	0.2	0.005	—	0.050
THPFOS	53 (4)	51 (5)	1.1	0.07	—	—
PFDS	39 (9)	72 (4)	0.2	0.04	—	0.084
PFHxA	82 (3)	80 (2)	0.7	0.1	—	—
PFHpA	82 (2)	84 (4)	0.3	0.1	—	—
PFOA	89 (2)	82 (4)	0.4	0.01	0.5	0.209
PFNA	95 (2)	77 (2)	0.2	0.005	—	—
PFDA	56 (5)	43 (27)	0.1	0.008	—	0.014
PFUnDA	47 (7)	38 (3)	0.2	0.005	—	0.008
PFDoDA	41 (11)	39 (5)	0.5	0.005	—	—
PFOSA	47 (4)	34 (0)	0.1	0.007	—	—

Abbreviations: —, below the detection limit; CV, coefficent of variation. Values for detection limit and blank concentration are from multiple experiments.

a Not including eventual blank concentrations.

b Mean ± 3 SD of detected signal from injecting 10 μL of a procedural blank.

**Table 3:** Levels (ng/mL) of seven PFCs in matched milk and serum samples from 12 primiparous Swedish women, 2004.

	PFHxS	PFOS	PFOSA	PFOA	PFNA	PFDA	PFUnDA
Serum							
No. > LOD	12	12	9	12	12	12	12
Range	1.8–11.8	8.2–48.0	< 0.10–0.49	2.4–5.3	0.43–2.5	0.27–1.8	0.20–1.5
Mean	4.7	20.7	0.24	3.8	0.80	0.53	0.40
SD	2.9	10.5	0.16	1.0	0.55	0.41	0.35
Median	4.0	18.7	0.19	3.8	0.63	0.43	0.28
Milk							
No. > LOD	12	12	8	1^a^	2	0	0
Range	0.031–0.172	0.060–0.470	< 0.007–0.030	< 0.209^b^–0.492	< 0.005–0.020	< 0.008	< 0.005
Mean	0.085	0.201	0.013	NA	0.017	NA	NA
SD	0.047	0.117	0.009	NA	NA	NA	NA
Median	0.070	0.166	0.010	NA	NA	NA	NA
M:S	0.02:1	0.01:1	0.07:1	0.12:1	0.01:1	NA	NA
CV	28	38	67	NA	52	NA	NA

NA, not applicable.

a Eleven additional samples were above the detection limit (0.01 ng/mL) but the blank level was > 50% of the detected concentrations (blank level 0.209 ng/mL).

b Blank level.

**Table 4:** Levels (ng/mL) of 5 PFCs in composite milk samples from primiparous Swedish women.

Region	Year of collection	PFHxS	PFOA	PFNA	PFOS	PFOSA
Uppsala	1996	0.037	< 0.209^a^	0.028	0.209	< 0.007
Uppsala	1997	0.030	< 0.209^a^	< 0.005	0.207	< 0.007
Uppsala	1998	0.040	< 0.209^a^	< 0.005	0.219	< 0.007
Uppsala	1999	0.044	< 0.209^a^	< 0.005	0.213	< 0.007
Uppsala	2000	0.028	< 0.209^a^	0.019	0.191	< 0.007
Göteborg	2001	0.028	< 0.209^a^	< 0.005	0.258	< 0.007
Uppsala	2002	0.051	< 0.209^a^	< 0.005	0.194	< 0.007
Lund	2003	0.025	< 0.209	< 0.005	0.153	< 0.007
Lycksele	2003–2004	0.016	< 0.209	0.020	0.123	< 0.007

a Levels were above the detection limit (0.01 ng/mL) but the blank level was > 50% of the detected concentrations (blank level 0.209 ng/mL).
